# Prevalence of periodontal disease among mine workers of Zonguldak, Kozlu District, Turkey: a cross-sectional study

**DOI:** 10.1186/s12889-018-5304-1

**Published:** 2018-03-16

**Authors:** Murat İnanç Cengiz, Büşra Zengin, Murat İçen, Firüzan Köktürk

**Affiliations:** 1 Department of Periodontology, Faculty of Dentistry, Bülent Ecevit University, 67600 Kozlu, Zonguldak Turkey; 2 Department of Endodontics, Faculty of Dentistry, Kocaeli University, Kocaeli, Turkey; 3 Department of Dentofacial Radiology, Faculty of Dentistry, Bülent Ecevit University, Zonguldak, Turkey; 4 Department of Biostatistics, Faculty of Medicine, Bülent Ecevit University, Zonguldak, Turkey

**Keywords:** Coal mining, Occupational health, Periodontal diseases

## Abstract

**Background:**

Occupational injuries cause major health problems in all nations. Coal mining is one of the largest, oldest industries in the world. However, there is relatively little available literature concerning the health status of coal miners. The purpose of this work is to assess the prevalence of periodontal disease among coal miners and provide a basis for planning and evaluating the data from community oral health services.

**Methods:**

A cross-sectional study was conducted 106 men selected based on a stratified cluster sampling procedure. The study was performed among the mine workers of Zonguldak, Kozlu District, Turkey. The questionnaire prepared by the American Academy of Periodontology risk assessment test was used for the evaluation. The data were collected byWorld Health Organization (WHO) oral health assessment form, and clinical examination was conducted by the method recommended by the WHO oral health surveys. Statistical analysis was performed using SPSS software programme.

**Results:**

The overall prevalence of periodontal disease was found to be 96.2% and was determined by considering subjects with Community Periodontal Index scores of 1–4 as diseased and the healthy subjects comprised of a mere 3.8%. Furthermore, various disturbing or embarrassing work conditions were reported. Statistically significant differences were observed among the workers who brush their teeth daily and visit dental attendance within the last two years have better periodontal status than those of the others (*p* < 0.05).

**Conclusions:**

The present level of periodontal disease in coal mine workers is severe. Moreover, its distribution and severity are strongly influenced by host susceptibility and risk factors. The priority should be based on population strategy and primary prevention programmes to benefit the periodontal health by promoting self-care and oral hygiene.

## Background

Individuals can develop diseases in association with genetic factors and environmental exposure. Increasing industrial activity around the world has improved people’s standards of living, but these increased activities have also led to various occupational hazards [1]. This situation affects people’s general and oral health due to their exposure to dangerous occupational environments [2, 3]. The effects of the aetiological agents responsible for the oral findings concerning occupational diseases depend on the physical, chemical, and bacterial characteristics of these agents and their entrance ways into the body.

Oral health is an integral part of general health. The oral cavity establishes a connection between the environment and the body, creating a region that is prone to occupational diseases because it is directly exposed to various occupational pollutants. Oral disorders are directly related to people’s general health status. Occupational diseases that occur in the mining, metalwork, and chemical industries can also affect the periodontium and oral mucosa [2, 3].

Trends in periodontal diseases have seen rapid changes throughout the world [3, 4]. Periodontitis is one of the most widespread chronic diseases at a global scale. Due to an increase in tobacco use, periodontal diseases are one of the major challenges faced by countries such as Turkey. The role of personal risk factors, such as poor lifestyle and negative psychosocial conditions, has been said to play an important role in the aetiology of adult periodontitis [5]. Previous epidemiological studies on the prevalence and severity of periodontal disease have reported that periodontal health is worse in the developing countries than it is in the industrialized ones. The researchers found that the metal [6], metalwork [7, 8] and chemical industries [8, 9] can affect periodontal disease patterns, but no specific pattern was observed in our study, and the pattern of periodontal disease in mine labourers was comparable to that of the general population.

Coal mining is one of the oldest branches of industry in both Turkey and the rest of the world [3, 10, 11]. The Zonguldak coal mining industry comprises many miners [11]. Tens of thousands of miners work for the National Hard Coal Enterprises (TTK), and private-sector businesses were legally commissioned in 2000; approximately 3000 miners are known to be illegal. In Turkey, the TTK Labour Security Officer is responsible for the implementation and distribution of information on security laws concerning the regulation of coal mines.

Because of the exhausting physical nature of the coal mining business, female miners were not seen in the study population. Workers’ exposure to coal dust and other types of particulate matter make them more prone to systemic diseases than the general population is. Coal mining production occurs throughout the week; the workers are engaged in tedious work around the clock, working in three rotating shifts of 8 h each. The physically tedious work drives people to consume alcohol and tobacco [12]. These substances may lead to the deterioration of their oral health in terms of periodontal and oral mucosal disease [13, 14]. Moreover, because of its disruption of the regular circadian rhythm activity in the body, shift work may lead to several dangerous health conditions [3, 11, 15].

The work environment is a major factor for health determinants, especially considering that oral health is crucial to the general health and well-being of individuals [16–18]. There are many studies about the oral health status of workers in different sectors. Among Indian workers in coal mines, 55.6% had caries experience [17], and in the industrial sector, the occurrence of dental caries was 46.5% [16]. Another study with Brazilian workers in a textile industry showed that orofacial pain had a significant impact on the performance of labour activities (28.5%), with tooth ache affecting 25% of individuals and generating absenteeism in 11.6% of workers [19]. Cavalcanti et al. found a high index of decayed-missing-filling (DMFT) in a study of the oral health status of Brazilian textile industry workers [20]. A cross-sectional study of white-collar port workers in India found thatoral health had an impact on the quality of life of workers, but periodontal disease, though prevalent amongst the participants, had no significant impact on their daily activities [21]. Only one paper has been published so far concerning the oral health status of underground coal mine workers [3]. Thus, the purpose of the present study was to assess the prevalence of periodontal disease among these workers. A further objective was to use the data to provide a baseline for planning and evaluating the oral health status of miners working in this field.

## Methods

A cross-sectional study was carried out to assess the periodontal disease status of underground coal mine workers in Zonguldak, Turkey, coal mines. Zonguldak is located in the Western Black Sea region and is the oldest mine in Turkey. A total of 106 subjects were included in this cross-sectional study. The questionnaire prepared by the American Academy of Periodontology (AAP) was employed. It included questions about sociodemographic factors, oral health knowledge, and periodontal and systemic health. All underground coal mine workers were asked to complete the survey. According to the scores, the study population was classified into groups with low, medium and high risk for periodontal diseases. The AAP risk assessment test was used to evaluate this risk [22]. The questionnaire items covered the following topics, which were considered in the data analysis: age, frequency of dental attendance, tooth brushing and flossing frequency, presence of gum recession, tooth extraction history, presence of gum diseases, and smoking habits.

The study population comprised 106 men aged 17–52 years, with a mean age of 32 ± 2 years, who were interested in having a periodontal examination. The study group was made up of workers who satisfied the inclusion and exclusion criteria. The inclusion criterion was that workers were present on the day of examination. The exclusion criteria were as follows: (1) individuals suffering from any systemic illness, (2) individuals who were not willing to participate in the study, and (3) individuals with a habit of bruxism. Ethical clearance was obtained from the Bulent Ecevit University Ethical Committee. Permission was obtained from the mine association and owners of the coal mine.

The study population comprised 106 men aged 17–52 years; the sample was divided into age groups of 17–36 years and 37–52 years. Education level was categorized into two groups: primary school only and more than primary school. Information on the frequency of daily tooth brushing was collected using the following question: “How many times did you brush your teeth yesterday?” (times/day). Participants were also asked whether they had visited a dental clinic for a regular check-up in the year prior to the interview (yes or no).

Smoking status was divided into two categories: non-smokers and current smokers. Current smokers were defined as those who smoked 1 pack per day. Obesity was measured based on a standardized physical examination. The participants self-reported weight and height, and we calculated body mass index (BMI) according to the standard formula. Weight was categorized as non-obese or obese using BMI 30 (kg/m^2^) as a cut-off-value, and underweight was classified as BMI < 18.5 kg/m^2^. Fasting plasma glucose and white blood cell (WBC) counts were measured, and chest X-rays were obtained.

All mine workers in the selected study group were informed about the survey prior to the survey date, and all workers who were present on the days of the survey were included in the study. Special permission was obtained from the coal mine owners’ association authorities for the miners to participate. An intraoral examination was performed in natural sunlight, and an additional light source was used when necessary. Oral examination was carried out using mouth mirrors and community periodontal index (CPI) probes, as recommended by the World Health Organization (WHO) [23]. A type 1 intraoral examination was performed by a single examiner assisted by two dental assistants. To reduce inter-examiner variability and enhance agreement, the examiners were trained prior to the study at Bülent Ecevit University’s Faculty of Dentistry by an expert examiner (MIC, Associate Professor in Faculty of Dentistry, Department of Periodontology). The weighted kappa statistic was 90%. Periodontal status was recorded using five possible scores: 0, healthy; 1, bleeding on probing; 2, calculus; 3, shallow periodontal pockets; and 4, deep periodontal pockets [24].

The recorded data were analysed using the Statistical Package for the Social Sciences (SPSS; SPSS Inc., Chicago, IL, USA) software, version 19. Descriptive statistics included computation of frequency and percentages. The statistical test applied for the analysis was the chi-square test. For all tests, the confidence level and level of significance were set at 95% and 5%, respectively.

## Results

Table 1 describes the general profile of the study population. All the workers were male and insured. They were all rotating-shift workers. The majority of workers belonged to the youngest age group (17–36 years), and only 7 workers belonged to the oldest age group (> 47 years). All the workers had very low incomes (less than $450 per month). Dietary intake of total calories was low. They were all underweight to normal weight (18.5 to 26 kg/m^2^).

**Table 1:** Demographic data, oral hygienepractices, tobacco and education

Variables	Levels	No.of subjects	Percentage
Age	17–36 years	84	79. 2
	37–52	22	20.8
Education			
Primary school	1–5 years	30	28.3
Middle school	6–8 years	26	24.5
High school	9–11 years	50	47.2
Years of working	1–5 yeras	57	53.8
	6–18 years	49	46.2
Oral hygiene aid	Finger	79	74.5
	Toothbrush	27	25.5
Tooth brush frequency	once a day	27	25.5
	2 3 times/week	79	74.5
Using material	None	37	35
	Water	42	40
	Toothpaste	27	25
Tobacco habits	Non-smokers	44	41.5
	currentsmokers (1 pack/day)	62	58.5
Alcohol habits	None	–	–
Working shift pattern	Rotating	106	100
Frequency of dental attendance within the last two years	yes	40	37.7
	no	66	62.3

Most workers had had eight years of schooling. More than half were unskilled workers. A total of 58.5% of the workers had the habit of smoking (current smokers: 1 pack/day). They were not heavy smokers. The rest, only 44 (41.5%) workers, were non-smokers. Alcohol and chewing tobacco were not used by the workers.

Nobody had visited any dental service or a dentist regularly. Only a minority of the workers had visited a dentist in emergency conditions. While 74.5% of the workers claimed to brush their teeth 2–3 times a week, only 25.5% reported daily tooth brushing. Most workers used only water and their fingers for cleaning their teeth. Only four workers claimed to have teeth in good condition, while the great majority of the workers declared that their teeth were bad. Healthy gingival conditions were reported by only four workers. Most of the workers reported that they were in need of dental treatment. A majority of the workers claimed to have had a great deal of trouble in their lifetime with their teeth or gums, and these problems were related to the work environment. Various disturbing or embarrassing work conditions were reported frequently (Table 2). The results for the questions on function of the masticatory system are shown in Table 3. The majority of the workers reported using analgesics for headache or facial pain daily or weekly.

**Table 2:** Percentage of workers who reported having been exposed to various work conditions (*n* = 106)^*^

Work condition	Percentage
Temperature excessively warm	10
Temperature excessively cold	12
Excessive changes in temperature	21
Draft	15
Humidity	35
Dirtiness	45
Dust	72
Unpleasant smell	20
Smoke	15
Mess and litter	30
Stress from the lifting of heavy weights	60
Vibration	40
High noise level	45

^*^More than one selection

**Table 3:** Percentage of respondents with (daily) masticatory system symptoms (*n* = 106)

Symptom	*n* = 106	Percentage of subjecs
Pain	2	1.8
Clicking or grating in jaw joint	3	2.8
Tenderness of teeth	27	25.8
Tenderness/ fatigue in cheeks	2	1.8
Difficulties in opening mouth	2	1.8
Tooth mobility	11	10.4
Bleeding while brushing	36	35.2
Grinding of teeth	5	4.7
Locking of jaw	–	–
Tooth recession	18	17.1

Table 4 shows the characteristics of the subjects by periodontal status. The overall prevalence of periodontal disease (CPI score 1–4) was 96.2%, and only 4 (3.8%) were classified as healthy (CPI score 0). In the largest age group (17 to 36 years), CPI scores 1 to 2 were most common (90.4%) and least common in the 37- to 52-year-old group (81.8%). CPI scores of 3 to 4 were most common in the 37–52-year-old group (18.2%) and least common in the 17- to 36-year-old group (4.8%). The healthy subjects, a mere 4.8% of the participants, were all in the 17- to 36-year-old group; there were no healthy subjects in other age groups. These findings were numerically important but not statistically significant (*p* > 0.05). Furthermore, no statistically significant differences were found between educational levels (1 to 8 and 9 to 11 years), years of working or smoking habit and periodontal CPI score (*p* > 0.05). The only statistically significant differences we found were that the workers who brushed their teeth daily and visited dental offices within the last two years had better periodontal status than the others (*p* < 0.05).

**Table 4:** Characteristics of subjects by periodontal status

	Variables	Health	Scores 1–2	Scores3–4	×2	P-Volve
Age	17 - 36 years (*n* = 84)	4 (4.8%)	76 (90.4%)	4(4.8%)	5.4	*P* > 0.05
	37 - 52 years (*n* = 22)	**_**	18(81.8%)	4(18.2)		
Education	1 to 8 years (*n* = 56)	1 (1.8%)	50 (88.3%)	5(8.9%)	1.5	*P* > 0.05
	9–11 years (*n* = 50)	3 (6%)	44(88%)	3(6%)		
Years of working	1 to 5 years (*n* = 57)	4 (7%)	50(87.7%)	3(5.3%)	4.3	*P* > 0.05
	6-to 18 years (*n* = 49)		44(89.8%)	5(10.2%)		
Tooth brush	daily (*n* = 27)	4(14.8%)	21(77.8)	2(7.4)	12.2	*P* < 0.05
	2–3/ weeks(79)	–	73(92.4%)	6(7.6%)		
Smoking	Non smoker (*n* = 44)	4(9.1%)	37(84.1%)	3(6.8)	5.8	*P* > 0.05
	Smoker *n* = 62		57(91.9)	5(8.1%)		
Frequency of dental attendance within the last two years	yes (*n* = 40)	4(10%)	34(85%)	2(5%)	7.2	*P* < 0.05
	no(66)	–	60(90.9%)	6(9.1)		
Total	(*n* = 106)	4(3.8%)	94(88.7%)	8(7.5%)		

## Discussion

People’s standard of living has been enhanced by the expansion of industrial activity; however, industrial production has also caused many occupational hazards. Developments in technology have made professions easier in many ways, but at the same time, these developments have brought new occupational hazards [1, 3]. Although coal mining is one of the oldest industries in the world [2, 3], little research has been carried out on how it affects coal miners’ health. This study sought to address this gap by considering periodontal disease in a Turkish population of underground coal miners. The study area for this research is a rural area where most people belong to the lower socioeconomic class and have low educational status. The workers in the mines work for at least 10 h per day to earn 1300–1800 Turkish lira (less than $450) per month. They work in deep, open pits where the air is thick with dust from dry drilling. Unfortunately, there is insufficient safety equipment to protect workers from occupational injury; for this reason, the coal mines are also called “death pits”. It is estimated that half the mine workers are exposed to this dust continuously, and they develop various pulmonary diseases due to the absence of respiratory masks. Workers’ living conditions are at a substandard level.

There is not much information available on the occupational general and oral health status of mine workers. Due to this lack research, in the context of oral health, it is difficult to compile and compare the data obtained from different studies in comparable groups. In this research, the oral health of underground workers in Zonguldak, Turkey, was evaluated by performing a cross-sectional study. In the literature, only one study has focused on coal miners [3]. In the present study, the workers were in the age range of 17 to 52 years. Our study subjects were younger than those of Abbas et al. and comparable in age to those of Kumar et al., Solanki et al., and Duraiswamy et al. [3, 8, 24, 25]. In addition, in this study, most subjects had 8 years of schooling, and the rest had 11 years of schooling. Our findings were comparable to those of Kumar et al. and Solanki et al., but they contrasted with Abbas et al.’s results, where 49.7% of the participants were uneducated [3, 8, 24].

In this study, in accordance with the literature, it was determined that the oral hygiene of miners is not good. Only 25.5% of the workers reported daily tooth brushing. Only four (3.8%) workers claimed to have teeth in good condition, while the overwhelming majority declared that their teeth were bad. Most workers claimed to have had extensive trouble with their teeth or gums in their lifetimes, and these problems were related to the work environment. Our findings concerning tooth brushing frequency are comparable to those in the literature [8, 24, 25]. However, contrary to the findings of Abbas et al., 63.2% of our participants reported using toothbrushes and toothpaste to clean their teeth daily [3]. In the present study, the workers were tobacco users (58.5%), although they were not heavy smokers (1 pack/day). Only 44 (41.5%) did not use tobacco in any form, and none of the workers used chewing tobacco or alcohol. The results concerning tobacco use are comparable to those of the literature [2, 3, 7, 8, 24–26], but the lack of alcohol usage contrasted with most findings in the literature. This may have been because alcohol was forbidden in the mine and is expensive.

Various disturbing work conditions were frequently reported. Oral diseases not only cause pain but severely impair many individuals and can affect various aspects of life, including oral functions, appearance and interpersonal relationships [21]. Most workers claimed to have had a great deal of trouble in their lifetime with their teeth or gums, and these problems were related to the work environment. Most workers reported using various analgesic medications to treat headache or facial pain daily or weekly. Furthermore, 35.2% of the coal workers experienced bleeding while brushing their teeth, 10.4% had tooth mobility, and 17.1% had tooth recession. Our investigation showed that coal workers had rather poor dental conditions. However, this finding was explained by their irregular dental care habits, since none of the participants had been treated by dental services or made regular visits to the dentist in their adult lives. The survey also showed that most coal workers were aware of their poor dental health status, and many related their dental problems to the work environment. Poor work conditions, including exposure to dust, were reported. Grinding and clenching of teeth due to hyperactivity in the masticatory muscles is considered necessary for the development of pathological attention. The findings concerning self-reported disorders of the masticatory system were supported by clinical observations. To the best of our knowledge, no previous study can be compared with this part of our research; the results for the coal mine worker population are shown in Tables 2 and 3.

Mining workers’ oral hygiene was extremely poor. A minority (25.5%) of the workers used a toothbrush and toothpaste to clean their teeth. This poor oral hygiene of the workers also affected their periodontal condition in terms of the CPI, gingival bleeding, and/or periodontal pockets. Healthy gingiva was observed in only 3.8% of the workers. The overall prevalence of periodontal disease was 96.2% (88,7% gingivitis and 7.5% periodontitis). This was determined by considering subjects with CPI scores of 1–4 as diseased and those with a score of 0 as healthy. All these findings were comparable to those of Abbas et al., Kumar et al., and Salonki et al., in which 94.4%, 98.25%, and 95.1% of subjects had an unhealthy periodontium, with healthy gingivae only observed in 5.6%, 1.75%, and 4.9% of subjects, respectively [3, 8, 24]. The low prevalence of periodontitis and the harshness of the working conditions may because the population was skewed towards a younger age.

No statistically significant differences were observed between age groups, educational levels (1–8 and 9–11 years), years of working, or smoking habits and periodontal CPI scores. Some differences between groups were numerically important, but not statistically significant (*p* > 0.05). These findings may have been due to the small sample size and the similarity of the variables to each other. Differences were observed between the workers who brushed their teeth daily and had visited dental offices within the last two years (who had better periodontal status) and the others (*p* < 0.05).

There is limited information regarding occupational estimates concerning oral health. In the present study, the prevalence of periodontal disease was found to be high among coal mine workers. However, we are aware of some possible methodological limitations: This study was conducted in only one mine in one city, and the sample size was small. Because this was a cross-sectional study, no cause-and-effect associations could be made. Nevertheless, we think that this research will lead to future studies with broader populations. More research should be carried out with a larger sample size to better characterize the oral problems faced by mine workers.

## Conclusions

In the present study, the prevalence of periodontal disease was high in coal mine workers. Preventive medical and dental services should be provided for this urgent-need group through the establishment of community health centres. Effective oral health education and promotion workshops should be organized for underground mine workers. The workshops should be aimed at changing the perception that oral health is disparate to general health, and emphasis should be placed on the importance of oral health and its relationship to general health.

## Abbreviations

AAP: American academy of periodontology
BMI: Body mass index
CPI: Community periodontal index
TTK: National hard coal enterprise
WBC: White blood cell
WHO: World Health Organization
